# ID 162 - Medicamentos Recomendados em Protocolos Clínicos e Diretrizes Terapêuticas para Doenças Raras

**DOI:** 10.5327/2237-9622.2025.v34s1.162

**Published:** 2025-11-25

**Authors:** Anna Carolina dos Santos Ramalho, Adriane Lopes Medeiros Simone, Bruna Bento dos Santos, Stéfani Sousa Borges, Daniela Oliveira de Melo

**Keywords:** protocolos, doenças raras, incorporação, SUS, forma farmacêutica, via de administração

## Abstract

**Introdução:**

Para abordar a temática de tratamento de doenças raras é necessário considerar a especificidade dessas doenças, a individualidade dos pacientes e o funcionamento do Sistema Único de Saúde em sua totalidade. Nesse contexto, uma das respostas à movimentação social e institucional por mais atenção e cuidado integrado para pacientes com essas condições são os Protocolos Clínicos e Diretrizes Terapêuticas (PCDT) para doenças raras. Para além da disponibilização dos medicamentos, o cuidado aos pacientes com doenças raras precisa considerar a complexidade do tratamento e as necessidades de cada paciente, principalmente no que diz respeito ao manejo e ao acompanhamento de medicamentos, exigindo clareza na descrição do tratamento indicado. O objetivo desse trabalho foi identificar e analisar os medicamentos recomendados nos PCDT de doenças raras e verificar a situação regulatória de cada especialidade indicada, disponibilidade de bula, presença na Lista de Medicamentos Referência da Agência Nacional de Vigilância Sanitária (Anvisa), além de verificar a padronização na nomenclatura, de acordo com as normas exigidas para descrição de Formas Farmacêuticas e Vias de Administração.

**Método:**

Considerando os documentos vigentes até 3 de novembro de 2022, foram realizadas buscas manuais nos sites da Comissão Nacional de Avaliação de Tecnologias em Saúde (Conitec) e do Ministério da Saúde, para identificar apenas PCDT com uma lista formal de medicamentos, conforme a Portaria SAS/MS n.° 375/2009. A plataforma RedCap® foi utilizada para criar um formulário de extração padronizado e coletar dados dos PCDT, incluindo a portaria e ano de publicação, fármacos recomendados, posologia, esquema de administração e forma farmacêutica. Após a coleta, foi verificado o status de referência dos medicamentos e consultadas as bulas na Agência Nacional de Vigilância Sanitária (Anvisa) para confirmar o registro, a concentração e a forma farmacêutica. Com essas informações, foram identificados os PCDT voltados para doenças raras.

**Resultados:**

Representando 42,6% (43) de todos os PCDT publicados pelo Ministério da Saúde, os PCDT de doenças raras preconizam o uso de 104 princípios ativos, descritos conforme as orientações e as diretrizes estabelecidas, com poucos erros relacionados à informação de via de administração (28; 26%) e descrição da forma farmacêutica (22; 12%), que impactam no entendimento do corpo multidisciplinar que acompanha esses pacientes. Além disso, entre as 180 especialidades farmacêuticas recomendadas nos PCDT, 16 (8,8%) apresentam situação regulatória irregular, enquanto 82 (45,5%) constam na Lista de Medicamentos Referência da Anvisa.

**Conclusão:**

A maioria das especialidades farmacêuticas recomendadas nos PCDT de doenças raras contém informações corretas de via de administração e forma farmacêutica. No entanto, foram identificados alguns erros de descrição e falta de registro regulatório para alguns medicamentos. Essa análise destaca a necessidade de regularização e padronização da descrição de administração dos medicamentos, para garantir maior segurança e eficácia no cuidado dos pacientes com doenças raras, considerando que nessa área os tratamentos disponíveis são escassos.

